# Artificial Intelligence Models for Predicting Triage in Emergency Departments: Seven-Month Retrospective Comparative Study of Natural Language Processing, Large Language Model, and Joint Embedding Predictive Architectures

**DOI:** 10.2196/83318

**Published:** 2026-03-10

**Authors:** Edouard Lansiaux, Ramy Azzouz, Emmanuel Chazard, Amélie Vromant, Eric Wiel

**Affiliations:** 1 Emergency Department Lille University Hospital Lille France; 2 Centre Antipoison, Lille University Hospital Lille France; 3 ULR 2694-METRICS, Lille University Lille France; 4 Department of Public Health, EA 2694, Lille University Lille France; 5 Emergency Department, Hôpital Pitié-Salpêtrière, Assistance Publique des Hôpitaux de Paris Paris France

**Keywords:** triage, emergency department, artificial intelligence, natural language processing, large language model, Joint Embedding Predictive Architecture, clinical decision support, FRENCH scale

## Abstract

**Background:**

Triage errors in emergency departments (EDs), including undertriage and overtriage, pose significant risks to patient safety and resource allocation. With increasing patient volumes and staffing challenges, artificial intelligence (AI) integration into triage protocols has gained attention as a potential solution.

**Objective:**

This study aims to develop and compare 3 AI models—natural language processing (NLP), large language model (LLM), and Joint Embedding Predictive Architecture (JEPA)—for predicting triage outcomes according to the French Emergency Nurses Classification in Hospital (FRENCH) scale and to assess their performance relative to nurse triage and clinical expert consensus.

**Methods:**

We conducted a retrospective analysis of prospectively collected data from adult patients triaged at Roger Salengro Hospital ED (Lille, France) over 7 months (June-December 2024). Three AI models were developed: TRIAGEMASTER (NLP with Doc2Vec + MLP), URGENTIAPARSE (LLM with FlauBERT + Extreme Gradient Boosting [XGBoost]), and EMERGINET (JEPA with variance-invariance-covariance regularization). Of 73,236 ED visits, 657 (0.90%) had complete audio recordings and structured data. Data were split 80:20 into training and validation sets with stratification. Gold-standard labels were established by senior clinician consensus (minimum 5 years of ED experience). The primary outcome was concordance with the gold-standard FRENCH triage level, assessed using weighted κ, Spearman correlation, F1-score, area under the receiver operating characteristic (AUC-ROC) curve, mean absolute error (MAE), and root mean square error (RMSE). Secondary analyses evaluated Groupes d’Etude Multicentrique des Services d’Accueil (GEMSA) prediction and performance by input data type.

**Results:**

URGENTIAPARSE demonstrated superior performance, with a composite z score of 2.514 compared with EMERGINET (0.438), TRIAGEMASTER (–3.511), and nurse triage (–4.343). URGENTIAPARSE achieved an F1-score of 0.900 (95% CI 0.876-0.924), an AUC-ROC of 0.879 (95% CI 0.851-0.907), a weighted κ of 0.800 (*P*<.001), a Spearman correlation of 0.802 (*P*<.001), an MAE of 0.228, and an RMSE of 0.790. Exact agreement was 90.0%, with near-agreement (+1 or –1 level) of 92.8%. However, training showed perfect accuracy (1.0) with poor validation performance (~0.5), indicating overfitting. EMERGINET achieved moderate performance (F1-score=0.731, AUC 0.686), while TRIAGEMASTER and nurse triage performed poorly (F1-score=0.618 and 0.303, respectively). For GEMSA prediction, URGENTIAPARSE maintained superiority (κ=0.863, Spearman=0.864, *P*<.001). Class 1 (highest acuity) was underrepresented (4/657, 0.61%), limiting undertriage risk assessment.

**Conclusions:**

The LLM-based architecture (URGENTIAPARSE) demonstrated the highest accuracy for ED triage prediction among the tested models, outperforming traditional NLP, JEPA, and current nurse triage practices. However, severe overfitting, extreme selection bias (657/73,236, 0.90%, inclusion), a monocentric design, and sparse high-acuity representation limit clinical applicability. Before deployment, the model requires regularization, external validation across diverse EDs, prospective testing, and comprehensive safety evaluation, particularly for undertriage detection. Integration of AI triage support systems shows promise but demands rigorous validation, bias mitigation, and transparent uncertainty quantification to ensure patient safety.

## Introduction

Triage in emergency departments (EDs) is vital for prioritizing patients by severity, directly affecting outcomes, efficiency, and resource use. In busy or understaffed settings, accurate triage is essential to ensure safe and equitable care.

In France, the French Emergency Nurses Classification in Hospital (FRENCH) triage scale has become a standardized reference tool for emergency medical services [1]. This 6-level urgency stratification system (1=immediate life-threatening to 6=nonurgent) was designed to promote consistency and reproducibility. However, in practice, its application often varies [2], shaped by disparities in nurse experience, time pressure, interpretive inconsistencies, and fatigue. These factors lead to 2 key errors: undertriage, in which serious cases are underestimated, risking delays in care and potentially preventable mortality; and overtriage, in which minor cases are overprioritized, leading to crowding and inefficient use of resources [3].

Against this backdrop, artificial intelligence (AI) has emerged as a promising complement to traditional triage [4]. AI systems in emergency medicine can process diverse data, operate continuously, and provide consistent and transparent decisions; however, their success over the past decade has been mixed.

Early AI triage systems relied on natural language processing (NLP) to extract clinical meaning from text, using hand-crafted rules or statistical models [5]. Although these early systems were effective, they lacked the ability to understand context, nuance, or rare cases. Advances emerged with large language models (LLMs) such as Bidirectional Encoder Representations from Transformers (BERT) and its French version, FlauBERT [6]. These transformer-based models provided deep contextual understanding. When combined with boosting algorithms such as Extreme Gradient Boosting (XGBoost), LLMs formed hybrid architectures that balanced high performance with interpretability [7], 2 pillars of clinical deployment. More recently, Joint Embedding Predictive Architectures (JEPA) have introduced a third paradigm. JEPA models do not predict outcomes directly; instead, they embed both inputs and targets (eg, triage categories) into a shared latent space. Using techniques such as energy minimization and variance-invariance-covariance regularization (VICReg) [8,9], these models can uncover subtle cross-modal patterns. However, their complexity poses ongoing challenges for interpretability, an essential requirement in medical contexts.

Few studies have compared AI models under consistent conditions [10,11]. Most focus on narrow tasks, rely on limited or noisy data, and are often developed outside the French health care system, reducing their relevance for users of the FRENCH scale. Furthermore, recent work has highlighted concerns regarding judgment biases in triage [11,12], hallucinations in LLMs [13], and the need for robust calibration and uncertainty quantification in clinical AI systems [14].

This study aims to address this gap. We conducted a retrospective analysis of triage encounters at the Centre Hospitalier Universitaire (CHU) Lille ED, one of France’s major academic hospitals [15]. Using rich clinical data and nurse-patient dialogues, 3 AI models—TRIAGEMASTER, URGENTIAPARSE, and EMERGINET—were developed to predict FRENCH triage levels. These models were evaluated against expert consensus to identify the best-performing system and to assess their clinical suitability and limitations.

## Methods

### Study Design

This study was designed as a retrospective, observational, monocentric analysis conducted in the Adult Emergency Department of the CHU Lille, France. The ED is a high-volume academic tertiary care facility serving a diverse patient population, with approximately 73,000 adult visits annually. The investigation covered a continuous 7-month period from June 1, 2024, to December 31, 2024. This time frame was selected to capture seasonal variations in ED activity and to ensure a sufficiently large and heterogeneous dataset.

### Ethical Compliance

The study adhered to strict French and European ethical standards, with approval and registration from the Comité d’Éthique de la Recherche en Santé, Health Data Hub filing (project number 18605502), and Commission Nationale de l’Informatique et des Libertés (CNIL) Méthodologie de Référence-004 compliance [16]. Audio recordings were collected with patient information at triage. Personal data were present before anonymization and underwent rigorous deidentification, including removal of names, dates, identifiers, and geographic information smaller than the department level, with transcript verification performed by 2 independent reviewers. As this was a retrospective analysis of deidentified data, individual informed consent was waived in accordance with French regulations. The study followed the Transparent Reporting of a multivariable prediction model for Individual Prognosis Or Diagnosis (TRIPOD)-AI guidelines (see Multimedia Appendix 1). Owing to patient confidentiality, the full dataset and code are not publicly available; only the code used to define the gold standard is shared (see Multimedia Appendix 2).

### Population and Inclusion Criteria

The study included adult patients (≥18 years) evaluated in the Lille CHU ED with complete structured clinical data and recorded nurse-patient audio interviews. Exclusion criteria were minors (<18 years), legally protected adults, patients who declined data use (n=2), and cases with incomplete recordings, corrupted files, missing structured data, or failed transcription quality checks (n=22).

### Data Acquisition and Preprocessing

The study relied on 2 primary data sources. First, structured clinical data were extracted from the ResUrgences ED management software (version 2024.1.148; Berger-Levrault). Variables collected included age, sex, vital signs (systolic and diastolic blood pressure, heart rate, temperature, oxygen saturation, and supplemental oxygen requirement), pain score (visual analog scale 0-10), time of presentation, comorbidities, chief complaint category, the initial FRENCH triage decision, and the Groupes d’Etude Multicentrique des Services d’Accueil (GEMSA) scale (corresponding to patient outcome after diagnostic evaluation and treatment in the ED) [17].

Unstructured data from nurse-patient triage interviews were audio-recorded using standardized digital recorders and manually transcribed by trained personnel, with accuracy verified by 2 independent reviewers. Transcripts were linked using unique patient identifiers and time stamps, anonymized in accordance with CNIL guidelines, and securely stored on Health Data Hub–approved servers for modeling. Recording participation was limited to volunteer nurses (n<10) during their regular shifts, introducing potential selection bias in conversation style and patient characteristics.

### Comparison of Included Versus Excluded Cohorts

To assess selection bias, we compared demographic and clinical characteristics between included patients (n=657) and the broader ED population (n=72,579) during the study period. The included cohort had a similar mean age (42.6 vs 43.2 years, *P*=.68), sex distribution (330/657, 50.2%, male, *P*=.67), and vital sign distributions. However, included patients had slightly lower acuity (mean nurse FRENCH 3.4 vs 3.1, *P*=.02) and higher admission rates (15.5% vs 12.3%, *P*=.03), likely reflecting volunteer nurse participation patterns and recording feasibility during less chaotic triage encounters (Table 1).

**Table 1:** Patient demographics, admission characteristics, vital parameters, and triage distributions.

Characteristic		Overall	Training	Validation
Age (years), mean (SD)		42.6 (19.7)	42.5 (19.8)	43 (19.5)
**Sex, n (%)**				
	Male	330 (50.2)	264 (50.2)	66 (50.4)
	Female	327 (49.8)	262 (49.8)	65 (49.6)
**Admission time, n (%)**				
	Night (9 PM-6 AM)	211 (32.1)	169 (32.1)	42 (32.1)
	Morning (6 AM-2 PM)	241 (36.7)	193 (36.7)	48 (36.6)
	Afternoon (2 PM-9 PM)	205 (31.2)	164 (31.2)	41 (31.3)
_**Chief complaint, n (%)**_				
	_Trauma, n (%)_	127 (19.3)	101 (19.2)	26 (19.8)
	_Abdominal, n (%)_	102 (15.5)	81 (15.4)	21 (16.0)
	Cardiovascular	86 (13.1)	68 (12.9)	18 (13.7)
	Neurological	69 (10.5)	55 (10.5)	14 (10.7)
	ENT (ear-nose-throat)/stomatology	62 (9.4)	49 (9.3)	13 (9.9)
	Other^a^	211 (32.1)	172 (32.7)	39 (29.8)
**Comorbidities, n (%)**				
	None	496 (75.5)	398 (75.7)	98 (74.8)
	≥1 comorbidity	161 (24.5)	128 (24.3)	33 (25.2)
**Vital signs, mean (SD)**				
	Systolic blood pressure (mmHg)	143 (24)	143 (24)	143 (23)
	Diastolic blood pressure (mmHg)	83 (16)	83 (16)	83 (17)
	Heart rate (bpm)	89 (18)	89 (18)	89 (17)
	Temperature (°C)	37.5 (0.6)	37.5 (0.6)	37.5 (0.6)
	Pain score (0-10)	4 (3.6)	4 (3.6)	4.1 (3.7)
	Peripheral capillary oxygen saturation (SpO_2_, %)	97 (2)	97 (2)	97 (2)
^Supplemental O2 (L/min), mean (SD)^		0.04 (0.4)	0.04 (0.4)	0.03 (0.3)
_**Nurse triage (FRENCH^b^), n (%)**_				
	1 (immediate), n (%)	4 (0.6)	3 (0.6)	1 (0.8)
	2 (very urgent), n (%)	86 (13.1)	68 (12.9)	18 (13.7)
	3A (urgent, invasive)	0 (0)	0 (0)	0 (0)
	3B (urgent, noninvasive)	354 (53.9)	283 (53.8)	71 (54.2)
	4 (less urgent)	118 (18.0)	94 (17.9)	24 (18.3)
	5 (nonurgent)	95 (14.5)	78 (14.8)	17 (13.0)
**Disposition (GEMSA^c^), n (%)**				
	1	0 (0)	0 (0)	0 (0)
	2 (discharged)	509 (77.5)	407 (77.4)	102 (77.9)
	3 (short stay)	18 (2.7)	14 (2.7)	4 (3.1)
	4 (ward admission)	102 (15.5)	82 (15.6)	20 (15.3)
	5 (intensive care unit admission)	10 (1.5)	9 (1.7)	1 (0.8)
	Not specified	18 (2.7)	14 (2.7)	4 (3.1)

^a^Other complaints are genitourinary, gynecology/obstetrics, infectious, toxicology, ophthalmology, dermatology, psychiatry, pulmonary, and rheumatology presentations.

^b^FRENCH: French Emergency Nurses Classification in Hospital.

^c^GEMSA: Groupes d’Etude Multicentrique des Services d’Accueil.

### Gold-Standard Construction

A gold-standard triage label was established through independent review by 3 senior emergency physicians (minimum 5 years of postresidency ED experience, board-certified in emergency medicine) who were strictly blinded to the original nurse triage assignment, all AI model outputs, and patient outcomes (laboratory results, imaging, disposition, and diagnoses established after the index triage encounter). Each adjudicator independently reviewed the complete triage transcript, vital signs, and demographic data, and then assigned a FRENCH level based solely on information available at the time of triage.

Initial interrater agreement was substantial (Fleiss κ=0.72, 95% CI 0.68-0.76). For the cases with disagreement (155/657, 23.6%), adjudicators met for a structured consensus discussion without knowledge of which assignments were discordant. Final consensus was reached in all cases through majority voting after discussion. This gold standard served as the reference for model training and evaluation. To assess potential incorporation bias, we conducted a sensitivity analysis excluding cases in which the gold standard differed from nurse triage by more than 1 level (n=47), and found similar model performance rankings.

### Model Architectures and Training Pipelines

Three distinct AI models were developed, each representing a major class of current machine learning techniques. All models were implemented in Python 3.9 (Python Foundation) with PyTorch 1.12 and scikit-learn 1.1, and trained on NVIDIA A100 graphics processing units (40 GB VRAM), with training times of 2-4 hours per model.

### Leakage Safeguards and Hyperparameter Tuning

#### Overview

Critical measures were implemented to prevent data leakage. All preprocessing steps (Doc2Vec fitting, FlauBERT fine-tuning, and feature scaling) were confined strictly to the training folds, with transformations subsequently applied to validation data. Hyperparameter tuning was performed using 5-fold stratified cross-validation nested within the training set only, without accessing validation data. Random seeds were fixed (seed=42) to ensure reproducibility. Early stopping monitored validation loss with a patience of 10 epochs to prevent overfitting.

#### TRIAGEMASTER (NLP)

This model used paragraph vector embeddings (Doc2Vec, distributed memory model; vector dimension=100, window=5, min_count=2, epochs=40) to represent free-text input [18], concatenated with *z* score–normalized numerical features derived from structured data (age, sex encoded as binary, and all vital signs). The architecture consisted of a 3-layer feedforward neural network (input→128→64→6 output classes) with Rectified Linear Unit activations [19], dropout regularization (*P*=.32, applied after each hidden layer), batch normalization, and an L2 weight penalty (λ=1×10^–5^). Optimization used the Adam optimizer with an initial learning rate of 0.001, batch size of 32, and learning rate decay (factor=0.5, patience=5 epochs). The maximum sequence length was 512 tokens. Missing data (<2% of vital signs) were imputed using median values from the training set.

Classification probabilities were derived via softmax activation. CIs for the area under the curve (AUC) were calculated using the DeLong method [20]. Model performance was evaluated across the full range of predicted probabilities. Optimal probability thresholds were defined as the predicted probability that maximized the *F*_1_-score [21]. Inference latency averaged 47 ms per patient on production CPU hardware (Intel Xeon Gold 6248R).

#### URGENTIAPARSE (LLM)

Built on the FlauBERT language model (flaubert-base-cased, 137 million parameters) [6], this architecture fine-tuned contextual embeddings from patient complaints over 5 epochs with a learning rate of 2×10^–5^, and then extracted the [CLS (classification)] token embedding (768 dimensions). These embeddings were concatenated with normalized structured variables and fed into an XGBoost classifier (n_estimators=200, max_depth=6, learning_rate=0.1, min_child_weight=3, subsample=0.8, colsample_bytree=0.8, reg_alpha=0.1, reg_lambda=1.0, scale_pos_weight adjusted for class imbalance). This hybrid design enabled both interpretability and high-dimensional pattern recognition [22]. Feature importance was derived using Shapley Additive Explanations (SHAP) values [23], providing transparent model behavior traceable to individual tokens and vital signs. The maximum sequence length was 512 tokens with truncation. Class weights were inversely proportional to class frequencies to address imbalance. Inference latency averaged 312 ms per patient.

#### EMERGINET (JEPA)

This JEPA encoded textual and structured inputs into a shared latent space (dimension=256) through dual encoders (text encoder: FlauBERT base + 2-layer bidirectional Long Short-Term Memory with hidden size 128 [24]; structured encoder: 3-layer MLP with hidden sizes (64, 128, 256)). The loss function minimized contrastive energy between predicted and target class embeddings using cosine similarity, regularized with VICReg (variance weight=1.0, invariance weight=1.0, covariance weight=0.04) to enforce variance >1.0, invariance via mean squared error, and a near-identity covariance matrix [8]. Training used the Adam optimizer (learning rate=1×10^–4^, batch size=16, 100 epochs). Final classification was performed using nearest-neighbor matching in embedding space with temperature-scaled softmax (temperature=0.07). Inference latency averaged 189 ms per patient.

### Training and Validation Procedures

Each model was trained on 80% of the dataset (n=526) and validated on the remaining 20% (n=131), stratified by gold-standard triage level to maintain class balance (class proportions: 1=4/657, 0.61%; 2=86/657, 13.1%; 3B=354/657, 53.9%; 4=118/657, 18.0%; and 5=95/657, 14.5%; classes 3A and 6 had 0 cases). Hyperparameter grids were searched using 5-fold cross-validation on the training data only. All models were trained 3 times with different random initializations to assess stability; reported metrics are means across runs, with SDs <0.03 for all primary metrics. Training and validation loss curves were monitored to detect overfitting.

### Evaluation Metrics

#### Performance Metrics for Ordinal Outcomes

Model performance was assessed using both absolute error measures and classification agreement metrics. Unless otherwise specified, all *P* values were 2-tailed, with a significance threshold of α=.05. Given the ordered nature of the FRENCH scale, metrics that account for ordinality (weighted κ, Spearman correlation, and mean absolute error [MAE]) were prioritized.

#### MAE

This measures the average deviation between predicted and actual triage levels.



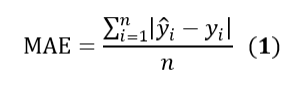



where 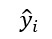 is the predicted value, *y_i_* is the true value, and *n* is the total number of cases.

#### Root Mean Square Error

This emphasizes larger misclassification errors:



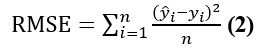



#### Weighted Cohen κ

This accounts for agreement beyond chance and penalizes larger discrepancies using quadratic weights:



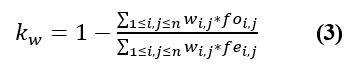



where *fo_i,j_* are observed frequencies, *fe_i,j_* are expected frequencies under independence, and *w_i,j_*=(*i* – *j*)^2^*/*(*k* – 1)^2^ with *k* classes. Significance was tested via bootstrap (10,000 iterations).

#### Spearman Correlation Coefficient

This measures the monotonic association between predicted and true ordinal ranks:

*r_s_*=[cov(*rg_X_,rg_Y_*)]/[*σrg_X_σrg_Y_*)] **(4)**

#### F1-scores

Macro *F*_1_-score reflects the unweighted average across all triage classes (treating each class equally), whereas micro *F*_1_-score is weighted by class frequency:

*F*_1_-score = (TP)/[TP+1/2(FP+FN)] **(5)**

where TP denotes true positive, FP false positive, and FN false negative. Class-specific *F*_1_-scores were calculated to assess performance in rare high-acuity classes.

#### Exact Agreement Percentage

This represents the proportion of predictions that exactly match the gold standard.

#### Near-Agreement Percentage

This represents the proportion of predictions within the +1 or –1 triage level of the gold standard.

#### Area Under the Receiver Operating Characteristic Curve

This summarizes discrimination across all classification thresholds, calculated using a one-versus-rest strategy for multiclass classification, with 95% CIs estimated via the DeLong method [20].

#### Composite z Score

This is an integrated performance indicator combining multiple metrics for ranking.

Score = *Z*(–MAE) + *Z*(–RMSE) + *Z*(*κ_w_*) + *Z*(*r_s_*) **(6)**

where *Z*(*X*) = (*X* – *µ_X_*)*/σ_X_* standardizes each metric to mean 0 and SD 1 across all models. Negative MAE and RMSE were used, so higher *z* scores indicate better performance for all components.

#### Multiclass Brier Score

This is the mean squared difference between predicted probabilities and one-hot encoded outcomes, providing global probabilistic accurac



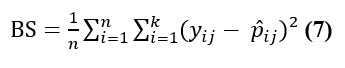



where 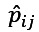 is the predicted probability for class *j* of case *i*, *y_ij_* is a binary indicator (1 if true class, 0 otherwise), and *k* is the number of classes.

### Asymmetric Error Analysis

Given the clinical importance of undertriage (predicting lower acuity than the true acuity, potentially delaying critical care), we analyzed error asymmetry by calculating the undertriage rate (proportion of predictions <true class) and the overtriage rate (proportion of predictions >true class). For classes 1-2 (highest acuity), we report sensitivity, specificity, positive predictive value, and negative predictive value separately, as undertriage in these categories poses the greatest patient safety risk.

### Calibration Analysis

Model calibration was assessed by grouping predicted probabilities into quantile-based bins (10 bins per class) and comparing mean predicted probabilities with observed frequencies. Reliability diagrams plotted mean predicted probability versus observed frequency for each bin, with perfect calibration represented by the diagonal. Expected calibration error and maximum calibration error were computed as measures of calibration quality. Additionally, risk distributions were visualized using density ridge plots stratified by true triage class and faceted by predicted class.

### Secondary Analyses

A secondary analysis compared actual versus AI-predicted GEMSA scores (patient disposition: 2=discharged home, 3=short stay unit, 4=admitted to ward, and 5=intensive care unit [ICU]) using models trained separately on nurse-recorded structured histories and full interview transcripts. Another analysis assessed model performance according to input data type (structured data only vs unstructured transcripts only). Both analyses used evaluation methods consistent with the primary analysis. All statistical analyses were performed in R version 4.2 (R Foundation) and Python 3.9.

## Results

### Population Characteristics

**Figure 1:**
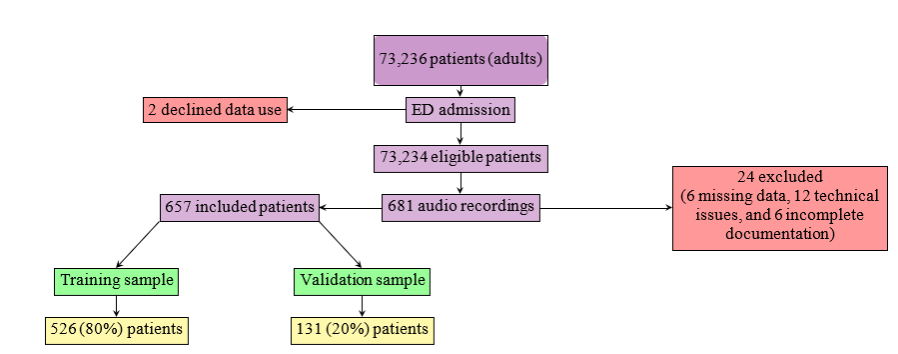
Patient flow diagram showing inclusion, exclusion, and dataset splitting. ED: emergency department.

During the inclusion period, 73,236 patients visited the ED of Roger Salengro Hospital. Two patients declined data use. Triage nurse interviews were recorded and transcribed for 681 (0.93%) patients. After excluding 24 cases (6 with missing data and 18 with transcription quality issues), 657 (0.90%) patients met the inclusion criteria (Figure 1).

Included patients had a mean age of 42.6 (SD 19.7) years and a median age of 38 years. The sample was gender-balanced (330/657, 50.2%, male). Most admissions occurred during morning hours (241/657, 36.7%, 6 AM-2 PM). The primary chief complaints were trauma (127/657, 19.3%), abdominal (102/657, 15.5%), and cardiovascular (81/657, 13.1%). One-quarter of patients (161/657, 24.5%) had 1 or more comorbidities, predominantly cardiovascular. Most patients were triaged as FRENCH 3B (354/657, 53.9%), with sparse representation in the highest acuity categories (class 1: 4/657, 0.61%).

### Training Performance and Overfitting Assessment

**Figure 2:**
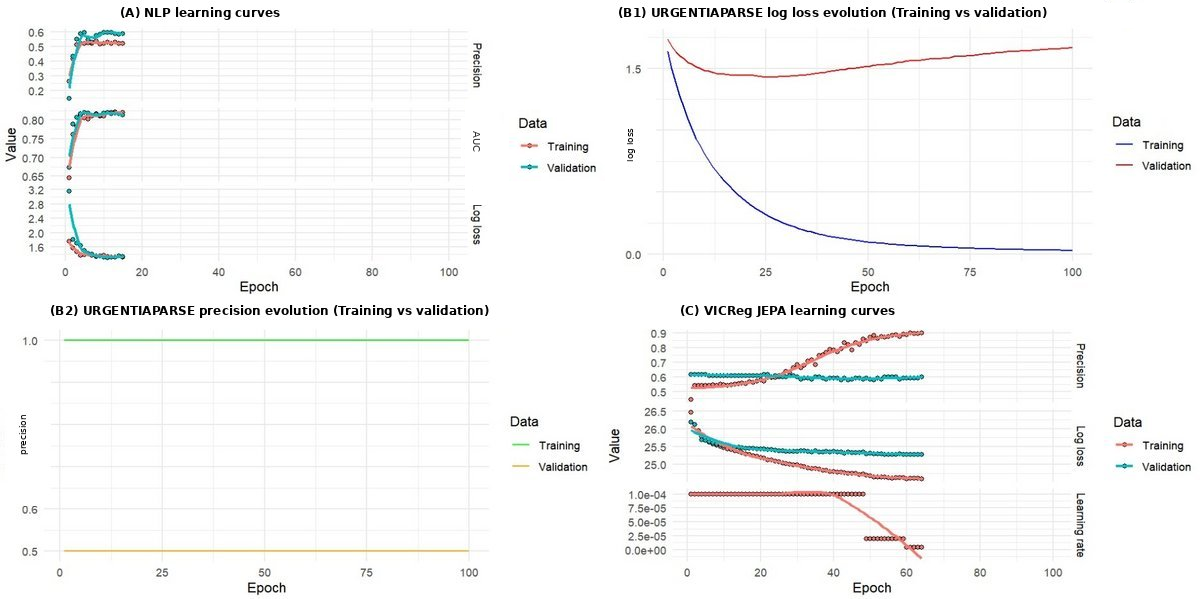
Learning curves for 3 artificial intelligence models. (A) TRIAGEMASTER shows stable learning with consistent training and validation performance. (B1) URGENTIAPARSE log-loss evolution demonstrates severe overfitting, with training loss approaching 0 while validation loss remains high. (B2) URGENTIAPARSE accuracy evolution confirms perfect training accuracy (1.0) but poor validation accuracy (0.5). (C) EMERGINET VICReg JEPA learning curves show moderate overfitting, with a gap between training and validation performance. JEPA: Joint Embedding Predictive Architecture; NLP: natural language processing; VICReg: variance-invariance-covariance regularization.

Over 100 training epochs, TRIAGEMASTER achieved a training accuracy plateau of approximately 75%, with validation accuracy similarly stable at 72%, while log loss decreased and stabilized for both sets, indicating reasonable generalization (Figure 2A).

URGENTIAPARSE exhibited severe overfitting, achieving perfect training accuracy (1.0) by epoch 20 but persistently poor validation accuracy (~0.5 throughout training), indicating memorization rather than learning generalizable patterns (Figure 2B.2). Training log loss approached 0, whereas validation loss remained high and flat at approximately 1.5, with an AUC plateau of 0.69 (Figure 2B.1). This discrepancy indicates that, despite strong performance on the training data, the model fails to generalize to unseen cases—a critical limitation for clinical deployment.

EMERGINET reached a training accuracy of 0.85, but validation accuracy remained at 0.60, indicating moderate overfitting (Figure 2C). Training loss declined steadily, whereas validation loss remained higher and stable, reflecting the performance gap.

### Overall Model Performance

URGENTIAPARSE (LLM) achieved the highest composite score (2.514), outperforming EMERGINET (JEPA, 0.438), TRIAGEMASTER (NLP, –3.511), and nurse triage (–4.343; Table 2). URGENTIAPARSE demonstrated strong discrimination, with a micro *F*_1_-score of 0.900 (95% CI 0.876-0.924), macro *F*_1_-score of 0.894 (95% CI 0.869-0.919), and AUC of 0.879 (95% CI 0.851-0.907). Agreement metrics were substantial: weighted κ of 0.800 (*P*<.001, 95% CI 0.761-0.839) and Spearman correlation of 0.802 (*P*<.001, 95% CI 0.768-0.832). Error metrics showed an MAE of 0.228 classes (approximately 1 triage level error every 4-5 patients) and an RMSE of 0.790. Exact agreement was 90.0%, with near agreement (+1 or –1 level) of 92.8%.

EMERGINET achieved moderate performance (micro *F*_1_-score=0.731, macro *F*_1_-score=0.747, AUC 0.686, κ=0.560, *P*<.001), whereas TRIAGEMASTER performed poorly (micro *F*_1_-score=0.618, AUC 0.642, κ=0.370, *P*<.001; Spearman=0.005, *P*=.95). Current nurse triage showed the lowest performance (micro *F*_1_-score=0.303, AUC 0.776, κ=0.080, *P*=.08; exact agreement=30.3%), likely reflecting the difference between real-time clinical judgment under time pressure and the gold standard established by senior physicians following comprehensive information review.

**Figure 3:**
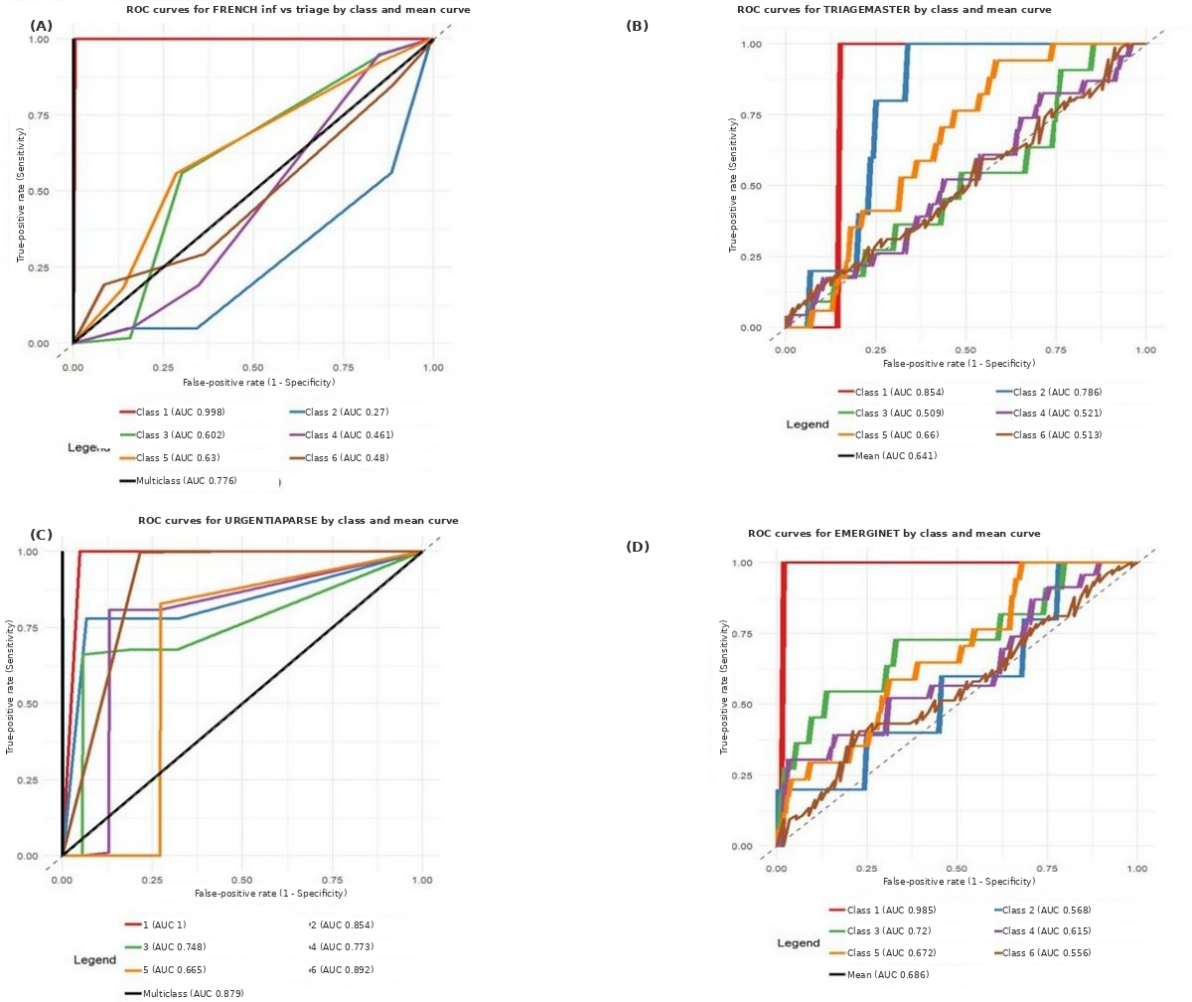
Receiver operating characteristic (ROC) curves for each triage class and the mean curve across all models. URGENTIA-PARSE (large language model) demonstrates superior discrimination for most classes. The results for class 1 (area under the curve [AUC] 1.0 for multiple models) should be interpreted cautiously due to extreme sparsity (n=4). (A) FRENCH inf vs triage — This panel shows ROC curves for a rule-based French triage reference system, where Class 1 achieves near-perfect discrimination (AUC 0.998) but most other classes perform poorly (AUC 0.27–0.63), yielding a modest multiclass AUC of 0.776. (B) TRIAGEMASTER — This panel displays the per-class ROC performance of TRIAGEMASTER, a structured NLP triage model, with moderate overall discrimination (mean AUC 0.641) and relatively homogeneous class-level AUCs ranging from 0.509 to 0.854, suggesting balanced but limited performance across triage levels. (C) URGENTIAPARSE — This panel presents URGENTIAPARSE's ROC curves, the best-performing model overall (multiclass AUC 0.879), with Class 1 achieving perfect separation (AUC 1.0) and strong performance across most classes, reflecting its superior ability to discriminate triage severity levels from free-text clinical notes. (D) EMERGINET — This panel illustrates EMERGINET's ROC curves, showing strong Class 1 discrimination (AUC 0.985) but more heterogeneous performance across the remaining classes (AUC 0.556–0.72), resulting in an intermediate mean AUC of 0.686.

Receiver operating characteristic (ROC) curve analysis (Figure 3) confirmed the superiority of URGENTIAPARSE across most triage classes, with particularly strong performance for classes 2, 4, and 5 (AUC >0.85). However, class 1 performance was perfect (AUC 1.0) because of extreme sparsity (n=4), limiting the reliability of this estimate. Performance for class 3 was lower (AUC 0.748), likely reflecting the combined representation of 3A (invasive procedures) and 3B (noninvasive), with no 3A cases in the dataset.

**Table 2:** Primary analysis comparing triage prediction performance across artificial intelligence models and nurse triage.

Model	Mean absolute error	Root mean square error	κ^a^	Spearman^b^	Micro *F*_1_-score	Macro *F*_1_-score	Exact	Near^c^	*z* score^d^	Area under the curve
URGENTIAPARSE	0.228	0.790	0.800	0.802	0.900	0.894	0.900	0.928	2.514	0.879
EMERGINET	0.401	0.979	0.560	0.602	0.731	0.747	0.820	0.860	0.438	0.686
TRIAGEMASTER	0.637	1.180	0.370	0.005	0.618	0.613	0.554	0.696	-3.511	0.642
Nurse triage	1.393	1.834	0.080	0.024	0.303	0.275	0.303	0.498	-4.343	0.776

^a^Weighted Cohen κ; for all κ values, *P*<.001 except nurse triage (*P*=.08).

^b^Spearman rank correlation; all *P*<.001 except TRIAGEMASTER (*P*=.95) and nurse triage (*P*=.76).

^c^Near agreement: proportion within the +1 or –1 triage level.

^d^Composite *z* score combining standardized mean absolute error, root mean square error, κ, and Spearman metrics (higher is better).

### Error Pattern Analysis

**Figure 4:**
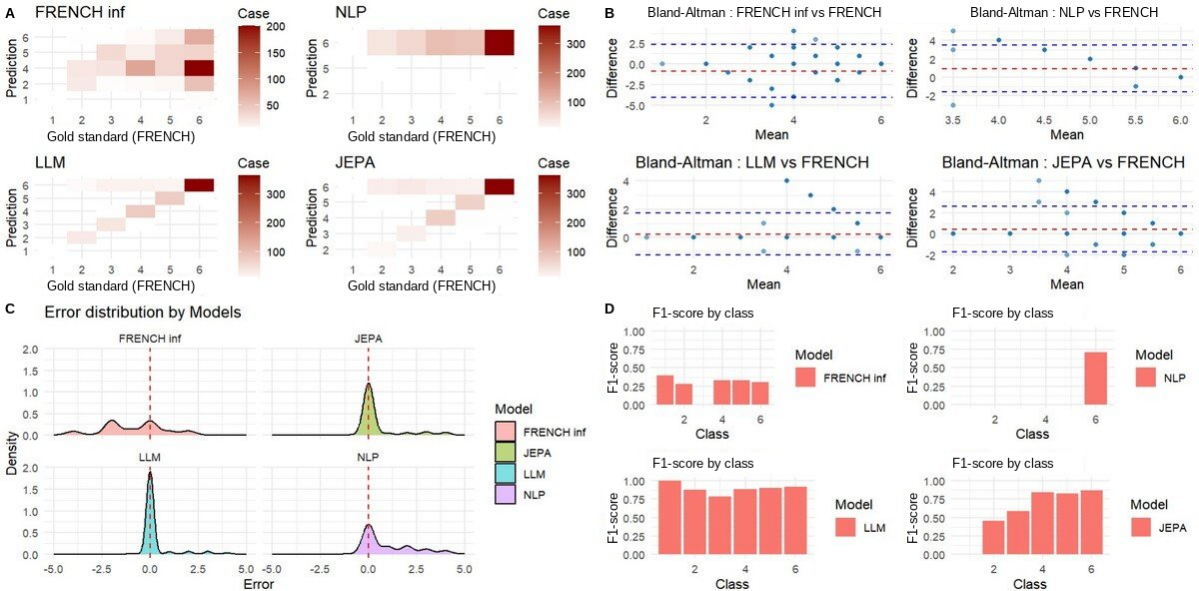
Comprehensive evaluation of four AI-based triage models against the FRENCH gold standard. (A) Confusion matrices comparing predicted versus gold standard triage levels (1–6) for FRENCH inf, NLP, LLM, and JEPA models. (B) Bland-Altman plots assessing systematic bias and limits of agreement between each model and the FRENCH gold standard. (C) Kernel density distributions of prediction errors illustrating the bias and precision of each model. (D) Per-class F1-scores quantifying the discriminative performance of each model across the six triage severity levels.

Confusion matrices revealed distinct error patterns (Figure 4, top row). URGENTIAPARSE and EMERGINET slightly overestimated acuity for lower triage levels (predicting higher urgency than true), indicating a clinically conservative bias. TRIAGEMASTER systematically underestimated acuity across most classes. Nurse triage showed scattered and inconsistent patterns, with frequent misclassification across nonadjacent categories.

Bland-Altman analysis (Figure 4, middle row) confirmed low, well-centered errors for URGENTIAPARSE (mean difference –0.02, 95% limits of agreement –1.58 to 1.54) and EMERGINET (mean difference 0.08, limits –2.05 to 2.21). TRIAGEMASTER showed larger deviations (limits –3.02 to 2.98), and nurse triage demonstrated extreme disagreement (limits –4.21 to 3.87).

Error distribution analysis (Figure 4, bottom left) showed that URGENTIAPARSE and EMERGINET had narrow, well-centered error distributions (SD <1.0), whereas TRIAGEMASTER and nurse triage exhibited broad, dispersed distributions, indicating less reliable predictions.

Class-specific *F*_1_-scores (Figure 4, bottom right) demonstrated that URGENTIAPARSE maintained consistently high performance across all represented classes (*F*_1_-score >0.75 for classes 2-5), whereas the other approaches showed variable and often poor class-specific performance, particularly for minority classes.

### Asymmetric Error and Undertriage Risk

Given the clinical importance of undertriage, we analyzed error directionality. URGENTIAPARSE had an undertriage rate of 4.6% (predicting lower acuity than true) and an overtriage rate of 5.4% (predicting higher acuity), reflecting a slight conservative bias favoring patient safety. For high-acuity classes (1-2, n=90), URGENTIAPARSE achieved a sensitivity of 87.8% (95% CI 79.2%-93.7%) and a specificity of 95.1% (95% CI 91.3%-97.6%), with a positive predictive value of 86.8% and a negative predictive value of 95.6%. However, the limited representation of class 1 (n=4) constrains the assessment of undertriage risk among the most critical patients. Future deployment would require prospective monitoring, with particular attention to undertriage events and associated patient outcomes.

### Calibration and Reliability

**Figure 5:**
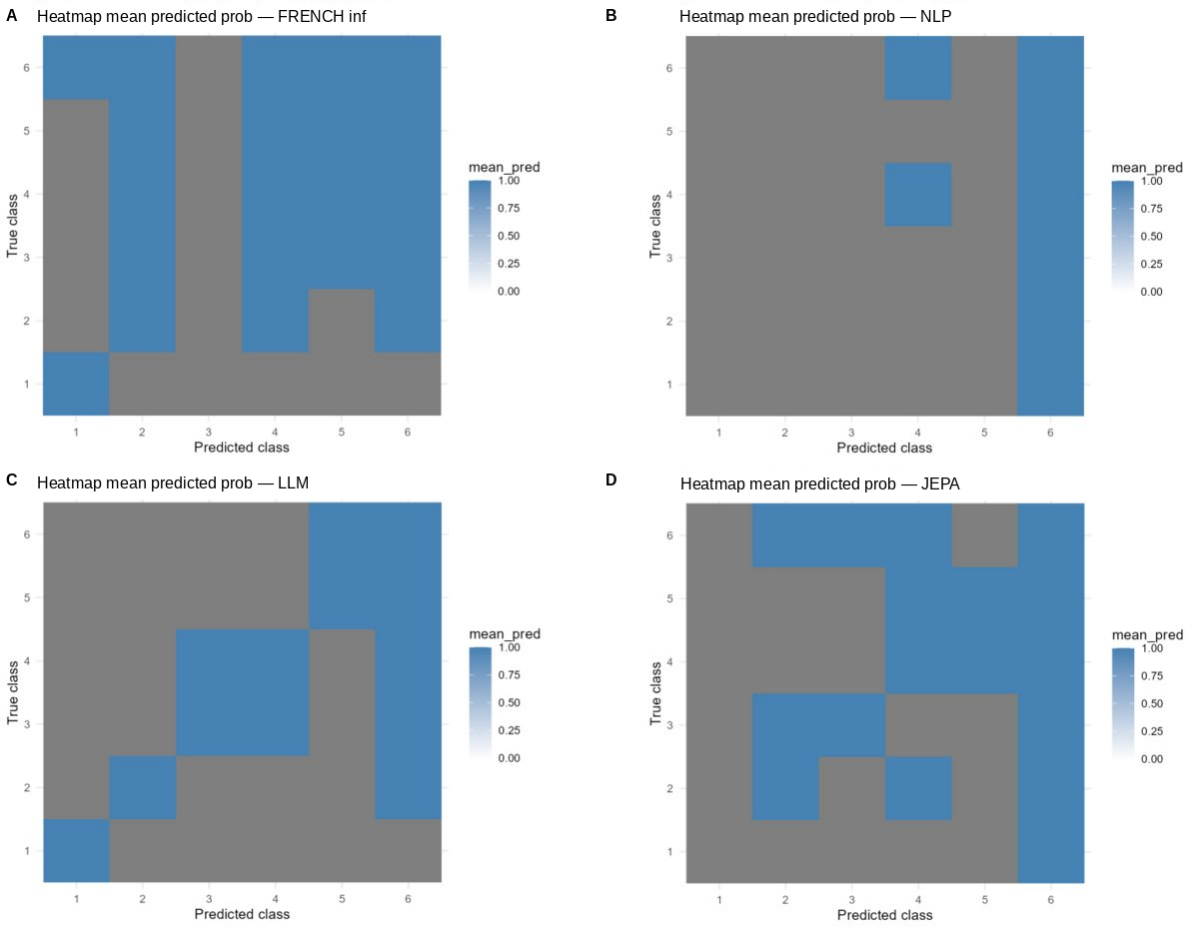
Mean predicted probability heatmaps for the four triage models. Each panel displays, for each true triage class (y-axis, 1–6), the mean predicted probability assigned to each predicted class (x-axis, 1–6), with blue intensity encoding probability magnitude (0–1): (A) FRENCH inf, (B) NLP, (C) LLM, and (D) JEPA — blue blocks concentrated along or near the diagonal indicate well-calibrated class-specific predictions. Perfect calibration would be indicated by high intensity only along the diagonal.

Multiclass Brier scores indicated superior probabilistic calibration for URGENTIAPARSE (0.142), compared with EMERGINET (0.385), TRIAGEMASTER (0.524), and nurse triage (0.693). However, detailed calibration analysis revealed heterogeneous reliability across classes (Figure 5).

Class-specific calibration analysis showed the following patterns:

- Class 1: Predictions were overconfident, with limited probability range coverage because of extreme sparsity.
- Class 2: Predictions were dominated by high-probability values (>0.8), indicating limited use of intermediate confidence levels.
- Class 3: Displayed a partially calibrated diagonal trend, but with inconsistencies across probability bins.
- Class 4: Demonstrated the most balanced calibration, with broader probability coverage and closer agreement between predicted and observed frequencies.

These findings highlight substantial class-specific differences and underscore the need for post hoc calibration methods (eg, temperature scaling or isotonic regression) before clinical deployment to ensure reliable probability estimates for risk stratification.

### Risk Distribution Analysis

**Figure 6:**
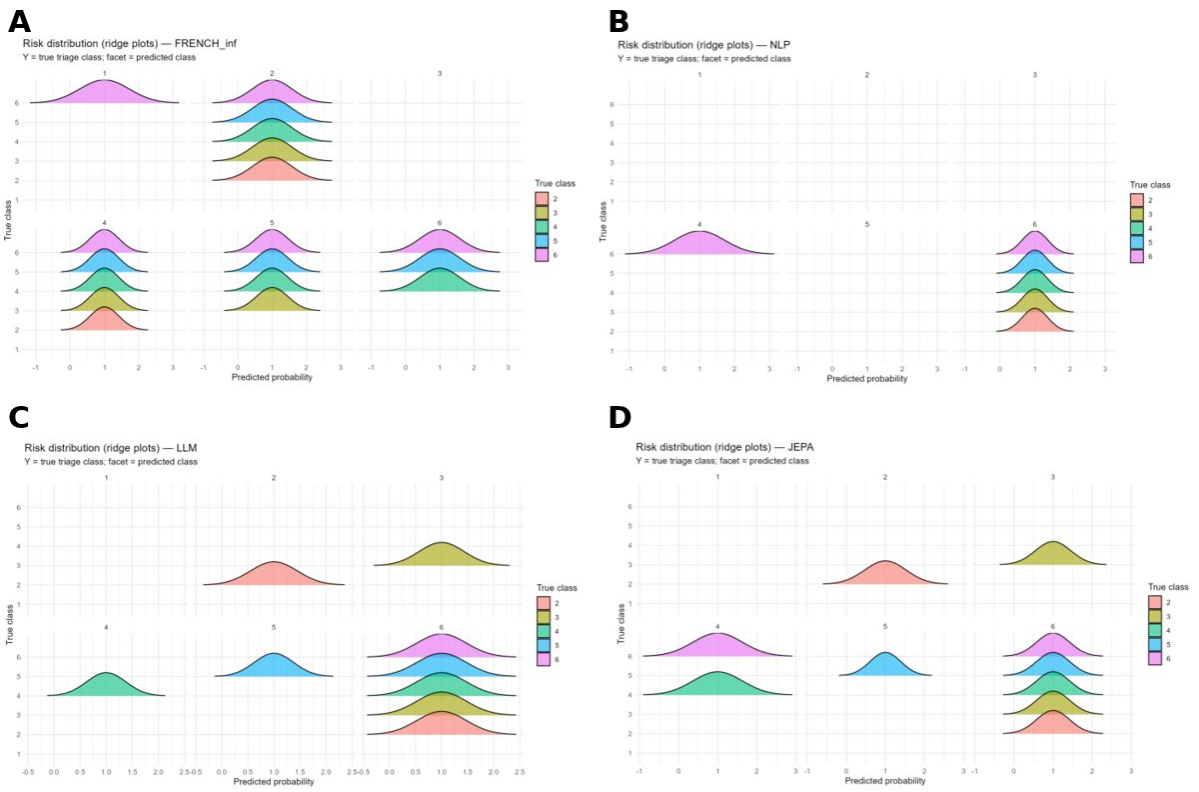
Risk score distributions by predicted triage class across models (ridge plots). For each model, predicted probability distributions are shown separately per predicted class (facets 1–6, columns) and colored by true triage class (2–6, see legend). (A) FRENCH_inf model (French clinical rule-based inference). (B) NLP model (natural language processing). (C) LLM model (large language model). (D) JEPA model (Joint Embedding Predictive Architecture). The x-axis represents the predicted probability; the y-axis represents the true triage class. Substantial overlap between distributions within a facet indicates high inter-class confusion for that predicted class.

Ridge plot analysis (Figure 6) revealed distinct behavioral patterns across approaches. Nurse triage demonstrated relatively consistent distributional patterns, with moderate overlap between adjacent classes and clear modal peaks, suggesting reasonable discrimination but substantial uncertainty.

TRIAGEMASTER exhibited variable performance, with notable gaps in certain class predictions (particularly panels 1 and 3), indicating an inability to consistently predict across all triage categories. Distributions were concentrated around specific probability values, suggesting either high confidence in limited scenarios or potential overfitting.

URGENTIAPARSE displayed markedly selective characteristics, with substantial gaps in class representation across prediction scenarios. Several triage classes appeared absent in certain combinations, suggesting that the LLM produced more selective predictions. When predictions were generated, distributions were tightly concentrated with distinct modal peaks, indicating high confidence. This sparse prediction pattern may reflect superior discrimination in clear-cut cases but raises concerns regarding coverage across diverse patient presentations. It may also suggest that the model effectively refrains from uncertain predictions—a behavior that requires explicit characterization and validation.

EMERGINET showed heterogeneous patterns, with some classes displaying broad, diffuse distributions and others exhibiting sharp, concentrated peaks, indicating variable predictive confidence across triage categories.

The distinct sparse pattern observed with URGENTIAPARSE warrants careful consideration for clinical implementation. Although it may indicate superior precision in unambiguous cases, the absence of predictions for certain class combinations presents challenges for comprehensive triage coverage. This pattern requires further validation to ensure adequate performance across all patient presentations, including edge cases and ambiguous scenarios.

### Secondary Analysis: GEMSA Prediction

URGENTIAPARSE substantially outperformed the other models in predicting patient disposition (GEMSA), achieving a composite *z* score of 2.473 versus –2.056 for TRIAGEMASTER and –4.399 for EMERGINET (Table 3). Performance metrics included an MAE of 0.082, RMSE of 0.402, weighted κ of 0.863 (*P*<.001), Spearman correlation of 0.864 (*P*<.001), micro *F*_1_-score of 0.957, and macro *F*_1_-score of 0.628. High exact agreement (95.7%) and near agreement (96.0%) further confirmed its accuracy and reliability.

**Figure 7:**
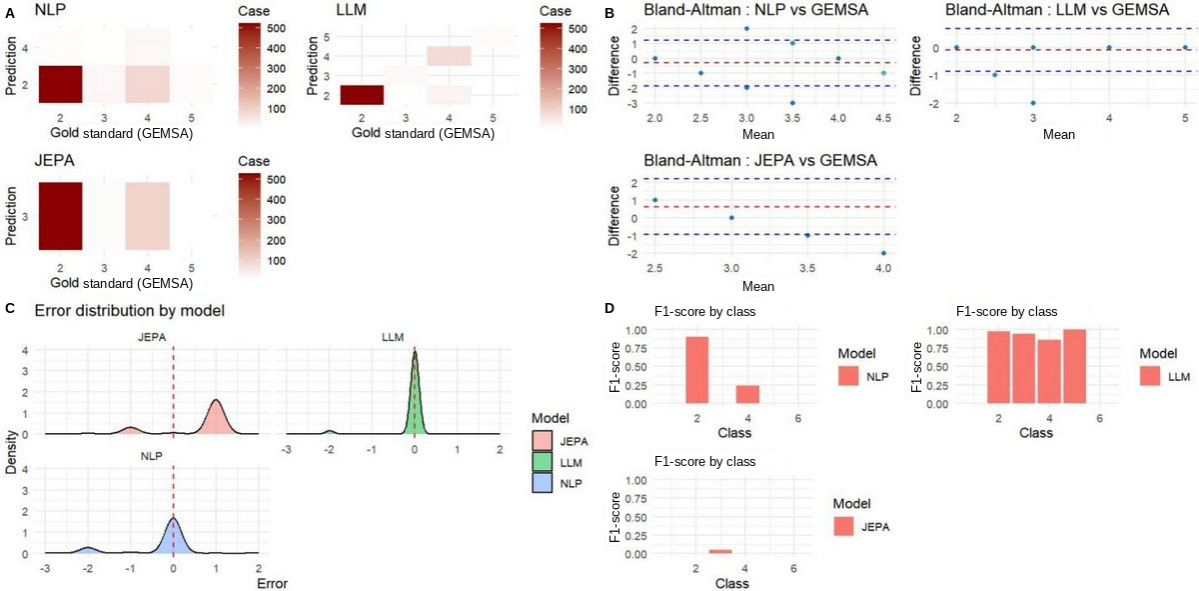
Evaluation of three AI triage models against the GEMSA gold standard. (A) Confusion matrices comparing predicted versus gold standard triage levels for NLP, LLM, and JEPA. (B) Bland-Altman plots showing bias and limits of agreement between each model and GEMSA. (C) Kernel density distributions of prediction errors illustrating model precision and systematic bias. (D) Per-class F1-scores quantifying discriminative performance across triage severity levels.

Confusion matrices (Figure 7, top) showed that URGENTIAPARSE produced highly concentrated predictions along the diagonal (accurate), EMERGINET demonstrated moderate accuracy, and TRIAGEMASTER displayed scattered predictions. Bland-Altman analysis (Figure 7, middle) confirmed strong agreement for URGENTIAPARSE (mean difference 0.04, limits –0.83 to 0.91), moderate agreement for EMERGINET (limits –2.12 to 1.96), and large deviations for TRIAGEMASTER (limits –2.51 to 2.25).

Error distributions (Figure 7, bottom left) showed that EMERGINET had small, tightly clustered errors; URGENTIAPARSE exhibited slightly broader but well-centered errors; and TRIAGEMASTER demonstrated wide, poorly centered distributions. Class-specific *F*_1_-scores (Figure 7, bottom right) revealed that URGENTIAPARSE achieved consistently high scores across the represented GEMSA categories, whereas EMERGINET and TRIAGEMASTER showed poorer and more uneven performance.

**Table 3:** Secondary analysis comparing artificial intelligence models for GEMSA^a^ (disposition) prediction.

Model	Mean absolute error	Root mean square error	κ^b^	Spearman^c^	Micro *F*_1_-score	Macro *F*_1_-score	Exact	Near^d^	*z* score^e^
URGENTIAPARSE	0.082	0.402	0.863	0.864	0.957	0.628	0.957	0.960	2.473
TRIAGEMASTER	0.364	0.863	0.185	0.240	0.811	0.198	0.811	0.840	–2.056
EMERGINET	0.988	1.009	0	0	0.027	0.009	0.027	0.985	–4.399

^a^GEMSA: Groupes d’Etude Multicentrique des Services d’Accueil.

^b^Weighted Cohen κ; URGENTIAPARSE and TRIAGEMASTER, *P*<.001; EMERGINET, *P*=.99.

^c^Spearman rank correlation; URGENTIAPARSE, *P*<.001; TRIAGEMASTER, *P*=.006; EMERGINET, *P*=.99.

^d^Near agreement: proportion within the +1 or –1 GEMSA level.

^e^Composite *z* score (higher is better).

### Impact of Data Modality: Structured Versus Unstructured Inputs

Analysis of input data type impact (Table 4) revealed that structured data (vital signs, demographics, and chief complaint codes) yielded slightly better performance than unstructured transcripts for URGENTIAPARSE (*z* score 1.806 vs 1.628) and TRIAGEMASTER (–4.790 vs –4.949), whereas EMERGINET showed minimal difference (–1.357 vs –0.993). These findings suggest that (1) vital signs and demographic data contain substantial triage-predictive information; (2) LLMs can extract meaningful patterns from both modalities; and (3) optimal performance likely requires multimodal integration. The relatively small performance gap between modalities for URGENTIAPARSE indicates that the transcript-based LLM captures much of the clinically relevant information, supporting the feasibility of voice-based triage assistance systems.

**Table 4:** Performance comparison across data modalities (structured vs unstructured inputs).

Model	Data type	Mean absolute error	Root mean square error	κ^a^	Spearman^b^	Micro *F*_1_-score	Macro *F*_1_-score	Exact	Near^c^	*z* score^d^
URGENTIAPARSE	Structured	0.213	0.748	0.821	0.826	0.903	0.898	0.903	0.928	1.806
URGENTIAPARSE	Unstructured	0.240	0.807	0.790	0.790	0.893	0.891	0.893	0.924	1.628
EMERGINET	Structured	0.510	1.247	0.420	0.559	0.807	0.551	0.807	0.836	–1.357
EMERGINET	Unstructured	0.516	1.240	0.430	0.560	0.791	0.539	0.791	0.843	–0.993
TRIAGEMASTER	Structured	0.916	1.557	0.057	0.160	0.572	0.190	0.572	0.712	–4.790
TRIAGEMASTER	Unstructured	0.991	1.629	-0.003	0.001	0.545	0.118	0.545	0.691	–4.949

^a^Weighted Cohen κ; *P*<.001 for all except TRIAGEMASTER unstructured (*P*=.97).

^b^Spearman rank correlation; *P*<.001 for all except TRIAGEMASTER (*P*>.20).

^c^Near agreement: proportion within the +1 or –1 triage level.

^d^Composite *z* score (higher is better).

## Discussion

### Principal Findings

This retrospective proof-of-concept study developed and compared 3 AI architectures for ED triage prediction using real nurse-patient interaction data and national triage standards. Of 73,236 ED visits over a 7-month period, 657 (0.90%) patients with complete audio recordings and structured data were included in the analysis. The LLM-based model (URGENTIAPARSE) demonstrated superior performance across multiple metrics (composite *z* score=2.514, *F*_1_-score=0.900, AUC 0.879, weighted κ=0.800, *P*<.001) compared with the JEPA-based EMERGINET (*z* score=0.438), the NLP-based TRIAGEMASTER (*z* score=–3.511), and current nurse triage practices (*z* score=–4.343). Secondary analyses confirmed the superiority of URGENTIAPARSE for disposition prediction (GEMSA) and its robustness across structured and unstructured data modalities. However, severe methodological limitations—particularly extreme selection bias, overfitting, sparse representation of high-acuity cases, and the monocentric design—preclude immediate clinical deployment and underscore the need for substantial additional validation.

### Comparison With Prior Work

Our findings align with and extend previous research on AI-assisted triage. The URGENTIAPARSE *F*_1_-score (0.900) substantially exceeds that of prior French ED triage models: Clinical BELGPT-2 achieved an *F*_1_-score of 0.62 [12], while external models such as Mistral 7B and Llama 3 8B demonstrated moderate performance, with larger models (Mixtral 8x7B) underperforming [11]. Our results also surpass those reported in the original FRENCH scale validation studies (AUC 0.83-0.86 for hospitalization prediction [1,25]) when applied by nurses, suggesting that AI has the potential to enhance triage accuracy.

However, unlike previous studies, we observed severe overfitting despite the use of regularization techniques. This likely reflects the extremely small effective sample size relative to model complexity (657 cases for the 137-million-parameter FlauBERT base), the high-dimensional input space (768-dimensional embeddings plus structured features), and substantial class imbalance with sparse representation of high-acuity cases. Recent studies on LLM hallucinations [13] and time-series forecasting [26] suggest potential mitigation strategies, including semantic entropy–based uncertainty quantification, prompt engineering, and backbone reprogramming—approaches that warrant investigation in future iterations.

Our calibration analysis revealed class-specific heterogeneity, consistent with recent findings on proper scoring rules for multiclass classification [14]. The observation that URGENTIAPARSE generates sparse, high-confidence predictions rather than well-distributed probability estimates suggests that the model may benefit from postprocessing approaches such as temperature scaling or Platt scaling to improve probability calibration before clinical implementation.

Prior studies have identified biases in triage decisions related to patient sex, nurse experience, and pain reporting [11,12]. Although our study did not explicitly assess these biases, the gold-standard consensus process and AI model evaluation framework established here could facilitate future bias detection studies. For example, systematic perturbation analyses, such as modifying patient sex references in transcripts while holding all other factors constant, could be conducted to evaluate potential model sensitivity to demographic attributes.

### Clinical Implications and Deployment Considerations

If validated prospectively across diverse settings, LLM-based triage support could enhance patient safety and operational efficiency through real-time decision support that highlights high-risk cases for immediate review; quality assurance by flagging discrepancies between nurse and AI triage for senior clinician verification; training tools that provide immediate feedback to novice nurses; and workload redistribution during surge periods by prescreening low-acuity cases. However, multiple critical barriers must be addressed before deployment.

The perfect training accuracy (1.0) with poor validation performance (~0.5) observed for URGENTIAPARSE indicates severe memorization rather than learning generalizable patterns. Potential solutions include substantial data augmentation, such as paraphrasing, synonym replacement, and back-translation for transcripts, combined with controlled perturbations of vital signs. More aggressive regularization strategies, including higher dropout rates, stronger L2 penalties, and techniques such as mixup or cutmix, may also help. Model simplification through adapter layers or low-rank adaptation (LoRA) fine-tuning of FlauBERT, rather than full model fine-tuning, could further reduce overfitting. In addition, ensemble methods that average predictions from multiple diverse architectures, transfer learning from related domains (eg, ambulance call triage or primary care urgency assessment), and active learning to selectively acquire cases with the highest model uncertainty all warrant investigation.

With only 4 (N=657) class 1 cases (0.61%) and 86 (13.1%) class 2 cases, we cannot reliably evaluate model performance for the most critical patients—those for whom undertriage could be fatal. Prospective deployment would therefore require conservative threshold selection that prioritizes overtriage over undertriage; mandatory senior clinician override for any AI-suggested downgrade relative to nurse triage; and real-time monitoring dashboards tracking undertriage events and associated patient outcomes, including ED length of stay, ICU admission, in-hospital mortality, and 72-hour return visits. In addition, automatic model deactivation should be triggered if the undertriage rate exceeds predefined safety thresholds.

Current probability estimates also require post hoc calibration—using methods such as temperature scaling, Platt scaling, or isotonic regression—to ensure reliable confidence estimates. Furthermore, implementing abstention mechanisms, whereby the model declines to issue a prediction when uncertainty exceeds a predefined threshold, may improve safety by deferring ambiguous cases to human judgment. Techniques from conformal prediction could additionally provide statistically rigorous uncertainty quantification with formal coverage guarantees.

Although SHAP values provide token- and feature-level explanations for URGENTIAPARSE predictions, clinicians require intuitive, actionable insights that align with real-world decision-making. Future work should therefore focus on generating natural language explanations—for example: “Triage level 2 suggested due to elevated heart rate (124 bpm), chest pain complaint, and description of respiratory distress.” Contrastive explanations could further enhance interpretability and trust, such as “If the heart rate were within the normal range, the predicted triage level would be 3.” Such counterfactual framing helps clinicians understand which features are most influential and how changes in patient presentation might alter the recommendation.

Real-time deployment requires inference latency under 5 seconds, a requirement met by the current 312-ms latency. However, seamless electronic health record integration without disrupting nurse workflow remains essential. An intuitive user interface that displays AI recommendations with confidence levels and key supporting features, combined with easy override mechanisms requiring mandatory documentation of the rationale, would facilitate adoption. Establishing feedback loops in which clinician corrections are logged for continuous model improvement completes the integration requirements.

AI triage support raises profound liability questions, with unclear responsibility among nurses, AI developers, and hospitals when undertriage occurs. Algorithmic bias is another concern, as model performance may vary by patient demographics, insurance status, or language. Privacy considerations regarding the secure handling of audio recordings and transcripts must be addressed, and equity requires ensuring that all patient subgroups receive accurate predictions. Comprehensive fairness audits, bias mitigation strategies, and clear governance frameworks must precede any deployment.

### Limitations

This study has multiple substantial limitations that constrain interpretation and generalizability across several domains.

The severe selection bias represents perhaps the most concerning methodological limitation. Only 657 of 73,236 (0.90%) ED visits had usable recordings, representing <1% of eligible encounters. Recordings were limited to volunteer nurses (fewer than 10) during feasible moments, likely excluding the highest-acuity cases requiring immediate intervention without time for a structured interview; chaotic shifts with multiple simultaneous arrivals; patients with altered mental status, language barriers, or communication difficulties; nurses uncomfortable with recording technology; and specific periods, such as overnight and weekends. The included cohort had slightly lower acuity than the broader ED population (*P*=.02), introducing systematic bias that limits generalizability to the real-world case mix. Future studies must implement systematic recording protocols that capture consecutive cases across all shifts, nurses, and patient types to obtain representative samples.

The sparse representation of the highest-acuity classes constitutes the most critical limitation for clinical deployment. Out of 657 cases, class 1 included only 4 (0.61%), class 2 included 86 (13.1%), and no class 3A or class 6 cases were observed. This extreme imbalance severely limits model training on high-risk cases, where undertriage has the greatest consequences. Reliable performance estimation for rare but critical categories becomes impossible, as CIs for class 1 metrics are extremely wide. The ability to detect undertriage patterns that could lead to preventable morbidity or mortality therefore remains fundamentally constrained. Generalization to settings with different acuity distributions also cannot be assessed. Class-specific performance metrics, particularly sensitivity for classes 1-2, have limited reliability and require validation in datasets with adequate representation across the full triage spectrum. Techniques such as the synthetic minority oversampling technique or focal loss to address extreme class imbalance should be explored in future work.

The severe overfitting observed for URGENTIAPARSE fundamentally limits real-world applicability. The model achieved perfect training accuracy (1.0), whereas validation accuracy remained approximately 0.5, clearly indicating that the model memorized training examples rather than learning generalizable triage patterns. This overfitting persisted despite dropout (*P*=0.3), L2 regularization (λ=1×10^–5^), and early stopping, suggesting that the effective sample size (n=657) is insufficient for the model complexity (137 million parameters in FlauBERT base). Although we report validation set performance rather than training set performance, the marked training-validation discrepancy signals a high risk of catastrophic failure on truly external data with different patient populations, nurse communication styles, or institutional protocols. Prospective external validation across multiple, diverse EDs is therefore essential before any consideration of clinical use. Potential solutions include more aggressive regularization, model simplification through adapter layers or LoRA fine-tuning, ensemble methods, or the collection of substantially larger training datasets, with a target of over 10,000 cases for stable LLM fine-tuning.

The monocentric design severely constrains external validity. Conducted at a single French academic tertiary care hospital, the results may not generalize to community hospitals with different patient demographics, resource levels, and triage processes. Rural or low-volume EDs, with different case mix and staffing patterns, represent an entirely different operational context. Non-French health care systems using alternative triage scales, such as the Emergency Severity Index, Canadian Triage and Acuity Scale, Manchester Triage System, or Australasian Triage Scale, would require complete model retraining and revalidation. Non-French language contexts would likewise necessitate retraining language models on appropriate corpora. EDs serving culturally, linguistically, or socioeconomically distinct patient populations may exhibit fundamentally different triage patterns. Multicenter validation across diverse settings—including academic and community hospitals, urban and rural locations, and multiple countries—is therefore required to assess transportability and identify factors that affect model performance.

The exclusion of repeat ED visits creates an important gap in our understanding of model performance. By including only index visits, we cannot assess model behavior in patients with frequent ED utilization, complex chronic conditions requiring a longitudinal perspective, or evolving clinical presentations across multiple encounters. Repeat visitors often present atypically and may benefit most from AI-assisted triage, yet they were systematically excluded from model development. This exclusion may bias performance estimates upward if repeat visitors are more difficult to triage accurately.

Several concerns regarding gold-standard construction could introduce incorporation bias. Adjudicators reviewed complete transcripts and vital signs with unlimited time for deliberation, in sharp contrast to real-time nurse triage conducted under time pressure and with incomplete information. This fundamental difference in decision-making context may overestimate AI-human agreement, as both the AI and gold-standard adjudicators had access to complete information unavailable to real-time nurses. Although blinded to outcomes, adjudicators were aware that they were establishing a research gold standard rather than making operational decisions with immediate consequences, which may have influenced risk tolerance and the willingness to assign higher acuity levels. The initial interrater agreement (κ=0.72) indicates substantial but not perfect consensus, with a 23.5% disagreement rate suggesting meaningful variability in expert interpretation even with complete information. The gold standard therefore reflects retrospective expert judgment, which may differ from optimal real-time triage, particularly when information available only after initial presentation is considered. A sensitivity analysis excluding cases with nurse-gold-standard discrepancies over 1 level yielded similar results, partially mitigating this concern but not eliminating it.

Limited independent validation of the FRENCH scale itself represents an often overlooked limitation. Although the FRENCH scale serves as the French national standard, independent validation studies remain limited beyond the original 2009 and 2019 publications [1,25]. The original validation reported an AUC of 0.858 for hospitalization prediction and moderate correlations with resource utilization. A 2019 validation study including 29,423 patients reported similar findings, with an AUC of 0.83 and moderate but significant correlations between triage level and resource utilization or testing [25]. Thus, even compared with FRENCH validation studies, the URGENTIAPARSE model demonstrates superior AUC performance relative to nurses who conducted triage in those studies. However, our gold-standard consensus process effectively establishes an “ideal FRENCH application” and does not validate whether FRENCH itself optimally predicts patient outcomes. Future research should therefore evaluate AI predictions against objective clinical end points, such as ICU admission, in-hospital mortality, 72-hour return visits, and length of stay, independent of triage scale assignment, to assess true clinical utility rather than agreement with an imperfect reference standard.

The lack of prospective validation and real-world testing fundamentally limits our ability to make clinical recommendations. This retrospective analysis cannot assess real-time inference performance or the integration challenges that inevitably arise during deployment. The impact on actual triage decisions and nurse acceptance remains unknown and could substantially affect real-world utility. Effects on patient outcomes, ED workflow, and resource utilization also cannot be determined from retrospective data. Model degradation over time, as patient populations, disease patterns, or institutional practices evolve through concept drift, represents a realistic threat that requires prospective monitoring. Rare failure modes and edge cases typically become apparent only during large-scale deployment involving thousands or tens of thousands of predictions. Unintended consequences, such as deskilling of nurses who become overly reliant on AI recommendations, or gaming of the system if performance metrics become targets, could further undermine potential benefits. Prospective stepped-wedge cluster randomized trials comparing AI-assisted versus standard triage across multiple sites, therefore, represent essential next steps before any clinical implementation can be responsibly considered.

The absence of cost-effectiveness and implementation analyses limits informed adoption decisions. We did not evaluate the computational infrastructure costs required for deployment, including graphics processing unit servers, maintenance, and updates, which may be substantial for resource-constrained hospitals. Training costs for nurses to use the system effectively also remain unknown and could be prohibitive if extensive instruction is required. The impact on triage time and ED throughput could be positive or negative, depending on implementation details. Cost per quality-adjusted life year gained represents the gold standard for health care economic evaluation, but requires long-term outcome data that are currently unavailable. Scalability challenges related to broad dissemination across diverse health care settings also remain unaddressed. Economic evaluation from a health system perspective is therefore essential for adoption decisions in the context of finite health care resources.

The absence of fairness and bias assessment represents a significant ethical limitation. We did not systematically evaluate whether model performance varies by patient demographics—including age, sex, race or ethnicity, insurance status, language, or socioeconomic status—potentially exacerbating health disparities. Prior work has identified sex-based and other biases in human triage decisions [11,12]; AI systems may perpetuate or amplify these biases if they are not explicitly addressed during development. If the model performs worse for already disadvantaged populations, deployment could worsen existing health inequities. Comprehensive fairness audits stratified by protected characteristics are therefore required before deployment, along with bias mitigation strategies if disparities are identified. Partnerships with ethicists, patients, and advocacy groups to define fairness metrics aligned with societal values should precede any clinical implementation.

Technological and data quality constraints affected the study throughout. Audio recording quality varied substantially, leading to transcription errors that may have affected model input quality in unpredictable ways. Equipment loss and technical failures resulted in 24 exclusions (3.5% of recorded cases), suggesting that real-world deployment would encounter similar challenges. Missing data for certain vital signs (<2%) required imputation using training set medians, which may have introduced bias. Real-world deployment would face these and additional challenges, necessitating robust handling of noisy, incomplete, or corrupted inputs with graceful degradation rather than catastrophic failure. The model’s behavior when confronted with poor-quality audio, incomplete vital signs, or technical failures remains unknown.

The lack of uncertainty quantification and explicit abstention mechanisms precludes safe clinical deployment. The model currently provides point predictions without well-calibrated CIs or explicit abstention when uncertainty is high. Clinical deployment requires principled uncertainty quantification—using methods such as conformal prediction, Bayesian approaches, or ensemble disagreement—to determine when deferral to human judgment is warranted. Without such mechanisms, the model may generate highly confident yet incorrect predictions in novel situations outside its training distribution. The sparse prediction patterns observed for URGENTIAPARSE may reflect implicit abstention behavior that should be explicitly characterized; however, we have not systematically examined when or why the model declines to issue predictions.

Limited interpretability persists despite SHAP analysis. Although SHAP values provide mathematical attributions indicating which input features most influenced each prediction, they may not align with clinical reasoning patterns or offer actionable insights for clinicians. A nurse or physician may not find numerical feature importance scores intuitive or helpful in understanding why the AI generated a specific recommendation. Natural language explanation generation that translates SHAP values into clinically meaningful statements remains underdeveloped. User studies assessing interpretability from the clinician perspective are therefore needed to determine whether these explanations facilitate, rather than hinder, clinical decision-making.

Regulatory and legal uncertainties cloud the path to clinical implementation. This research prototype has not undergone regulatory review, such as Conformité Européenne (CE) marking in Europe or Food and Drug Administration (FDA) clearance in the United States, which would likely be required for medical device classification of clinical decision support software. The regulatory pathway for AI-based triage support systems remains unclear and continues to evolve. Legal liability frameworks for AI-assisted triage are also poorly defined. When undertriage occurs with AI assistance, responsibility among nurses, AI developers, and hospitals remains ambiguous. These legal and regulatory uncertainties must be resolved before widespread deployment can proceed.

Finally, the absence of a continual learning framework limits long-term sustainability. Static models trained on historical data inevitably degrade as patient populations, diseases, and clinical practices evolve. Deployment would therefore require active monitoring of model performance in production; detection of performance degradation or concept drift; triggered retraining protocols when performance declines; and potentially online learning mechanisms to adapt to changing patterns while maintaining safety. We have not developed or validated any such infrastructure. Without continual learning capabilities, the model would become progressively obsolete, even if initially successful.

Despite these numerous and substantial limitations, this study provides important proof-of-concept evidence that LLM architectures can achieve high accuracy for ED triage prediction when evaluated using carefully collected retrospective data. The study also establishes a rigorous evaluation methodology that incorporates multiple complementary metrics and comprehensive error analysis, upon which future research can build. The limitations outlined above provide a detailed road map for the substantial additional work required before clinical translation can be responsibly considered, including the specific methodological improvements, additional studies, and infrastructure development necessary to support safe and effective deployment.

### Future Research Directions

Building on these findings and addressing the identified limitations, future research should prioritize several key areas. Trials across more than 10 diverse EDs—including academic and community hospitals, urban and rural settings, and varied patient demographics—with systematic consecutive case enrollment targeting more than 10,000 patients would enable assessment of external validity, detection of site-specific effects, and adequately powered subgroup analyses. A stepped-wedge cluster randomized design would allow causal inference regarding the impact on patient outcomes, including mortality, length of stay, ICU admission rate, and 72-hour return visits, while controlling for temporal trends.

Oversampling the highest-acuity encounters in classes 1-2 through deliberate recording protocols during trauma activations, stroke alerts, cardiac arrests, and other critical presentations would enable reliable detection of undertriage and robust safety evaluation. This targeted data collection would address one of the most important current limitations.

Systematic comparison of advanced overfitting mitigation strategies is also warranted. Such evaluation should include parameter-efficient fine-tuning approaches (eg, LoRA, adapters, and prompt tuning); aggressive data augmentation (eg, back-translation, paraphrasing, synonym replacement, and controlled vital sign perturbations); semisupervised learning leveraging unlabeled transcripts; multitask learning jointly predicting triage, disposition, resource utilization, and outcomes; transfer learning from related domains such as ambulance call triage or primary care urgency assessment; and ensemble methods combining diverse architectures with different inductive biases.

Systematic assessment of model behavior under a range of perturbations would strengthen confidence in its robustness. These should include adversarial attacks representing intentional gaming; distribution shifts due to seasonal variation, pandemics, or novel diseases; missing data resulting from incomplete vital signs, silent patients, or technical failures; transcription errors arising from speech recognition failures; and edge cases, such as rare presentations, complex comorbidities, or psychiatric crises. Developing certified robustness guarantees represents an important objective.

Stratified performance evaluation by protected characteristics—including age, sex, race or ethnicity, insurance status, language, and socioeconomic status—combined with detection of disparate impact, root cause analysis of identified biases, and rigorous testing of mitigation strategies (eg, reweighting, fairness constraints, or adversarial debiasing) would address equity concerns. Partnerships with ethicists, patients, and advocacy groups to define fairness metrics aligned with societal values are essential.

Development of natural language explanation generation, contrastive explanations that articulate how predictions would change if specific features differed, visualization tools highlighting salient features, and user interface optimization informed by clinician feedback would enhance interpretability. Human factors studies evaluating how AI recommendations influence decision-making—including risks of automation bias (overreliance) or dismissiveness (underreliance)—would further inform safe implementation.

Rigorous cost-effectiveness analysis from a health system perspective; budget impact modeling; implementation barrier assessment; stakeholder engagement with nurses, physicians, administrators, and patients; workflow redesign; training program development; and sustainment planning would support adoption. Qualitative studies exploring nurse and physician attitudes, concerns, and acceptance would help identify potential obstacles early.

Infrastructure for continual model performance monitoring, concept drift detection, triggered retraining protocols, online learning mechanisms, and transparent reporting to end users and regulators would maintain performance over time. Development of living systematic reviews tracking field performance as deployment expands would build the evidence base.

Exploration of synergies with AI-assisted diagnosis supporting downstream care decisions, predictive models for ED crowding and resource planning, automated documentation reducing nurse burden, and early warning systems for deterioration after initial triage could create an integrated ecosystem. Emerging work on AI transforming health care from acute care to early prevention [27,28] suggests potential for broader impact across the care continuum.

Collaboration with regulatory agencies, including the European Medicines Agency and FDA, to establish appropriate oversight frameworks balancing innovation with patient safety, defining clinical validation requirements, and clarifying liability and postmarket surveillance obligations for AI medical devices would facilitate responsible translation.

### Conclusions

This study demonstrates that LLM-based AI (URGENTIAPARSE) can achieve high accuracy for ED triage prediction, outperforming traditional NLP (TRIAGEMASTER), JEPA (EMERGINET), and current nurse triage practices when validated against a senior clinician consensus gold standard. With an *F*_1_-score of 0.900, AUC of 0.879, weighted κ of 0.800, and 90% exact agreement, the LLM model shows promise for enhancing triage consistency and potentially supporting clinical decision-making.

However, severe methodological limitations mandate extreme caution. Critical barriers to clinical deployment include severe overfitting, with perfect training accuracy but poor validation performance indicating an inability to generalize beyond the training data; extreme selection bias, with only a 0.9% (657/73,236) inclusion rate limiting representativeness; sparse high-acuity case representation, with only 4 class 1 cases precluding reliable undertriage risk assessment where stakes are highest; a monocentric design constraining external validity; retrospective analysis without prospective real-world validation; and absent economic, workflow, fairness, and regulatory analyses.

Before any consideration of clinical deployment, this AI triage support system requires large-scale prospective multicenter validation targeting more than 10,000 cases across diverse settings; comprehensive overfitting mitigation and model robustness enhancement; targeted high-acuity case collection enabling undertriage safety evaluation; fairness audits and bias mitigation strategies; post hoc calibration and uncertainty quantification with abstention mechanisms; interpretability enhancement with natural language explanations; human factors studies and workflow integration testing; economic evaluation and implementation planning; regulatory review and approval; and continuous performance monitoring infrastructure with concept drift detection.

While this proof-of-concept establishes technical feasibility and identifies the most promising architecture among the tested alternatives, the substantial gap between current evidence and safe clinical deployment must be acknowledged transparently. AI-assisted triage holds potential to improve patient safety, enhance consistency, reduce cognitive burden on nurses during surge periods, and support quality assurance—but realizing these benefits responsibly requires rigorous validation, thoughtful implementation, continuous monitoring, and humility about current limitations. This study provides a foundation and road map for that essential future work.

## Appendix

Multimedia Appendix 1 TRIPOD-AI fullfilled checklist.

Multimedia Appendix 2 Code for gold-standard.

## Abbreviations

AI: artificial intelligence
AUC: area under the curve
BERT: Bidirectional Encoder Representations from Transformers
CE: Conformité Européenne
CHU: Centre Hospitalier Universitaire
CLS: classification
CNIL: Commission Nationale de l’Informatique et des Libertés
ED: emergency department
FDA: Food and Drug Administration
FN: false negative
FP: false positive
FRENCH: French Emergency Nurses Classification in Hospital
GEMSA: Groupes d’Etude Multicentrique des Services d’Accueil
ICU: intensive care unit
JEPA: Joint Embedding Predictive Architecture
LLM: large language model
LoRA: low-rank adaptation
MAE: mean absolute error
NLP: natural language processing
RMSE: root mean square error
ROC: receiver operating characteristic
SHAP: Shapley Additive Explanations
TP: true positive
TRIPOD: Transparent Reporting of a multivariable prediction model for Individual Prognosis Or Diagnosis
VICReg: variance-invariance-covariance regularization
XGBoost: Extreme Gradient Boosting
